# Temporal and spatial distribution of lumpy skin disease outbreaks in Ethiopia in the period 2000 to 2015

**DOI:** 10.1186/s12917-017-1247-5

**Published:** 2017-11-06

**Authors:** W. Molla, M. C. M. de Jong, K. Frankena

**Affiliations:** 1 Quantitative Veterinary Epidemiology, Wageningen University & Research, Droevendaalsesteeg 1, 6708 PB Wageningen, The Netherlands; 2 Faculty of Veterinary Medicine, University of Gondar, P.O. Box 196, Gondar, Ethiopia

**Keywords:** Ethiopia, Lumpy skin disease, Time series, Spatial, Temporal, Forecast

## Abstract

**Background:**

Lumpy skin disease (LSD) is an infectious viral disease of cattle caused by a virus of the genus *Capripoxvirus*. LSD was reported for the first time in Ethiopia in 1981 and subsequently became endemic. This time series study was undertaken with the aims of identifying the spatial and temporal distribution of LSD outbreaks and to forecast the future pattern of LSD outbreaks in Ethiopia.

**Results:**

A total of 3811 LSD outbreaks were reported in Ethiopia between 2000 and 2015. In this period, LSD was reported at least once in 82% of the districts (*n* = 683), 88% of the administrative zones (*n* = 77), and all of the regional states or city administrations (*n* = 9 and *n* = 2) in the country. The average incidence of LSD outbreaks at district level was 5.58 per 16 years (0.35 year^−1^). The incidence differed between areas, being the lowest in hot dry lowlands and highest in warm moist highland. The occurrence of LSD outbreaks was found to be seasonal. LSD outbreaks generally have a peak in October and a low in May. The trend of LSD outbreaks indicates a slight, but statistically significant increase over the study period. The monthly precipitation pattern is the reverse of LSD outbreak pattern and they are negatively but non-significantly correlated at lag 0 (*r* = −0.05, *p* = 0.49, Spearman rank correlation) but the correlation becomes positive and significant when the series are lagged by 1 to 6 months, being the highest at lag 3 (*r* = 0.55, *p* < 0.001). The forecast for the period 2016–2018 revealed that the highest number of LSD outbreaks will occur in October for all the 3 years and the lowest in April for the year 2016 and in May for 2017 and 2018.

**Conclusion:**

LSD occurred in all major parts of the country. Outbreaks were high at the end of the long rainy season. Understanding temporal and spatial patterns of LSD and forecasting future occurrences are useful for indicating periods when particular attention should be paid to prevent and control the disease.

**Electronic supplementary material:**

The online version of this article (10.1186/s12917-017-1247-5) contains supplementary material, which is available to authorized users.

## Background

Lumpy skin disease (LSD) is an infectious viral disease of cattle caused by LSD virus of the genus *Capripoxvirus* and the disease often occurs as epidemics. It has spread from Zambia, where it was first observed in 1929 to most African countries (except Libya, Morocco, Algeria and Tunisia), Middle Eastern countries, and more recently also to European countries [1–5]. LSD can occur in diverse ecological zones from the very dry semi-desert, the wet and dry areas to the high altitude temperate areas [1].

LSD was introduced in Ethiopia, for the first time, through north-west (Gojjam and Gondar) in 1981 with subsequent introductions in the West (Wollega) in 1982 from Sudan and in the central part (Shewa) in 1983 [6]. After the introduction, the disease initially spread Eastwards, later to all directions and currently it has affected all regions and agro-climatic zones of the country [6–8]. The spread of LSD was enhanced by uncontrolled cattle movements, communal grazing and watering, and pastoralism [2, 7]. The poor animal health situation, inefficient prevention and control efforts in combination with late detection of the disease have further contributed to the spread of LSD in Ethiopia [2, 8].

In general, the temporal pattern of disease occurrence can be described with short-term, cyclical and seasonal, and long-term trends; time series analysis is a frequently used method to assess these temporal patterns [9]. The cyclical trends are associated with regular, periodic fluctuations in the level of disease occurrence. A seasonal trend is a special case of a cyclical trend, where the periodic fluctuations in disease incidence are related to particular seasons [9]. Seasonal variation in the occurrence of infectious diseases is a common phenomenon in both temperate and tropical climates. Seasonal changes in vector abundance are well-known causes of seasonality of vector-borne infections. A good knowledge on the seasonal variation of disease outbreaks has paramount importance for the understanding of the dynamics of the disease and in designing better control strategies [10].

Field observations and experimental studies indicate that blood feeding arthropods are involved as passive vectors in the transmission of LSD virus [2, 11]. The spread of LSD has been frequently associated with epidemics [12]. Epidemics of LSD occurred during the rainy season in which the arthropod vector populations are abundant while LSD incidence sharply drops during the dry and cold weather seasons [1, 13, 14]. Seasonal variation in the incidence of LSD outbreaks is common in Ethiopia in which it occurs most frequently between September and December [15]. Resurgence of the disease has been consistently associated with the high rainfall, emergence of large numbers of vectors and a low level of herd immunity [13, 16]. Epidemics of LSD were reported to recur at intervals of 5 or 6 years [13]. The reoccurrence of the disease in provisionally free area is possible when the infection is introduced into the population and the reproduction ratio (R), the average number of secondary cases caused by a single typical infectious individual, becomes greater than one [17].

Animal disease monitoring data is of fundamental importance to know the disease status of a country. In Ethiopia, the disease monitoring is mainly passive as most of the disease outbreaks reported to the federal veterinary services are based on clinical observations [18]. Monitoring of livestock diseases in the field is the responsibility of regional animal health services, regional veterinary laboratories and district animal health personnel. Disease investigations are generally conducted in response to reports of health problems from livestock owners. There is a regular follow up of disease outbreaks but the monthly livestock disease reporting rate is less than 47% which is below the required OIE (world organization for animal health) standards of at least 80% [19].

Assessing the spatial and temporal patterns is a prerequisite for guiding successful surveillance and control efforts in a country. Therefore, the objectives of this study were to evaluate the spatial and temporal distribution of LSD outbreaks and to forecast future patterns of outbreaks in Ethiopia based on data reported over the period 2000–2015.

## Methods

### Study area

Ethiopia is located in Eastern Africa bordering with Sudan, Eritrea, Djibouti, Somalia, Kenya, and South Sudan. It is a federation of nine member regional states (Tigray, Afar, Amhara, Oromia, Benshangul-Gumuz, Gambella, Southern Nations Nationalities and Peoples Region (SNNP), Harari, and Somali) and two city administrations (Addis Ababa and Dire Dawa). The regional states and city administrations are further divided into zones and the zones into woredas (districts), and the woredas into kebeles. As a whole there are about 15,000 kebeles (5000 urban dwellers associations in towns and 10,000 peasant associations in rural areas) in the country [20–22]. The country’s territory presents a diverse topography, ranging from 116 m below sea level at the Dallol Depression, in the East, to 4620 m above sea level on the Ras Dashen in the North and covers an area of approximately 1.1 million km^2^. Ethiopia is broadly divided into three climatic zones: “Kolla” (the hot lowland zone below 1500 m); “Weyna Dega” (mid highland zone between 1500 and 2400 m); and “Dega” (the cool highlands zone above 2400 m). Average daily temperature ranges from 20 °C to 30 °C. Rainfall ranges from 200 mm to 2000 mm per year. Ethiopia receives heavy rainfall in June, July and August and occasional showers in February and March. In general, the highlands of Ethiopia receive more rain than the lowlands [20, 21].

The total cattle population of the country is estimated to be about 56.71 million heads, mostly local breeds (98.7%); the remaining are hybrid (1.2%) and exotic breeds (0.1%) [23]. The livestock production system practiced in the country is usually extensive. In the highland and mid highland, it is highly integrated with crop production where cattle are primarily kept for traction purpose and to provide milk and meat as by-products. In the lowland, where no or little farming is practiced, pastoralists and agro-pastoralists keep cattle to provide mainly milk [23, 24].

### Outbreak and weather data

LSD is a notifiable disease and it is required that all occurrences of this disease be reported. LSD outbreak data were obtained from the Federal Veterinary Services Directorate of Ethiopia for the period 2000–2015. The records contained information on place, time, number of cases, number of deaths and number of animals at risk for each month. The reporting format enables calculation of the temporal and spatial distribution of LSD. An outbreak is defined as one or more bovines showing LSD symptoms in a specified geographical area (usually Kebele). During the 16 years period, no significant changes in operation of the veterinary organization that could have affected the level of reporting from the field were noted.

The LSD outbreak incidence was established at district (woredas, *n* = 683) level using the 16 years outbreak data. The mean LSD outbreak incidence in a district was calculated by summing all reported LSD outbreaks in a district over the study period and divide it by 16. The geographical distribution of LSD outbreaks over the 16 years was mapped by administrative zone using GIS software QGIS 2.2 (QGIS developer team, Open Source Geospatial Foundation, 2014). The spread of the epidemic was also shown using SPMAP (South Platte Mapping and Analysis Program, Stata 14) by superimposing the yearly outbreak data onto Ethiopian Woreda 2008 shape files in Microsoft power point program.

The monthly mean precipitations for the period 1999–2013 were obtained from the Global weather data for SWAT (Soil, Water, and Air Team) website. From a meteorological point of view, three seasons can be distinguished in Ethiopia; ‘Belg’ (February to May), ‘Kiremt’ (June to September) and ‘Bega’ (October to January). *‘*Kiremt’ is the main rainy season in which the magnitude of rainfall is highest as compared to the other seasons for many parts of the country [25].

### Data analysis

Data on the number of LSD outbreaks reported each month during the 16-year study period were analysed to detect temporal trends and seasonal effects. A simple inspection of the graph of the original LSD outbreak time series was employed to appreciate the presence of a clear long-term trend or seasonal effect. The existence of a long-term trend in LSD outbreaks was modelled by linear regression (STATA version 14) using the number of LSD outbreaks (or trend component of the outbreak) as dependent variable and month of the outbreaks as explanatory variable. Spectral analysis with SAS (Statistical Analysis Software) 9.4 was performed to detect seasonality and cyclical patterns in the LSD outbreak time series.

Decomposition of LSD outbreak time series was performed using package ‘TTR’ (Technical Trading Rules) in R software, to identify and estimate the three components of the temporal additive model: seasonality, long-term trend, and irregularity [26].

The time series were also seasonally differenced (i.e. deducting the 12 months earlier observation value from each observation value) first followed by first order trend differencing (i.e. deducting the preceding observation value from each observation value) according to the procedure described by Allard [27] and Coghlan [26] to make the series stationary (diff function in R). Next autocorrelation analysis (Autocorrelation function in R) was used to assess the seasonality of the differenced time series. The autocorrelation function (ACF) enables to test the significance of seasonality in a time series by examining the ACF correlogram at lags of 12 month intervals [28]. The ACF estimates the correlation between the number of outbreaks reported in a given month and the number of outbreaks reported in each of the previous 1 to 192 months. The autocorrelations and partial autocorrelations values of various lags were used for the selection of terms to be included in the initial autoregressive integrated moving average (ARIMA) model (autocorrelations and partial autocorrelations functions in R).

The Holt-Winters exponential smoothing technique as described by Coghlan [26] was applied to make short term (36 months) forecasts using package ‘forecast’ in R software. The possibility of improving the predictive model was evaluated by making a correlogram and carrying out the Ljung-Box test on the in-sample forecast errors for evidence of non-zero autocorrelations at lags 1 to 20. In this method the estimates of the parameters alpha, beta, and gamma represents the level, the slope of the trend component, and the seasonal component, respectively at the current time point. All the three parameters have values between 0 and 1. Parameter values that are close to 0 indicate that relatively little weight is placed on the most recent observations while forecasting future values.

Exponential smoothing methods are useful for making forecasts, but it does not take into account the correlations between successive values of the time series. However, a better predictive model can be made by taking correlations in the data into account. ARIMA models include an explicit statistical model for the irregular component of a time series that allows for non-zero autocorrelations in the irregular component. An ARIMA (1, 1, 1) x (1, 1, 1)12 model [26, 29] seemed a plausible model for the LSD outbreak stationary time series and this model was used to forecast the expected numbers of LSD outbreaks for a 36 month (January 2016 to December 2018) future time using the “forecast.Arima()” function in the “forecast” R package. Finally, it was investigated whether or not successive forecast errors of an ARIMA (1, 1, 1) x (1, 1, 1)12 models were correlated by making a correlogram and carrying out the Ljung-Box test.

The association between monthly rainfall and monthly LSD outbreaks was tested with Spearman rank test (Stata version 14).

## Results

### Geographical distribution and incidence of LSD outbreaks

During the period 2000–2015, LSD has been reported from all regional states (*n* = 9) and city administrations (*n* = 2) of Ethiopia. About 82% of the districts (*n* = 683) and 88% of the administrative zones (*n* = 77) in the country reported at least one LSD outbreak in this time period. In total 3811 LSD outbreaks were reported in Ethiopia during the study period (Additional file 1: Table S1). Most of these outbreaks were from Oromia (54.5%), Amhara (27.9%), SNNP (10.1%) and Tigray regional states (3.6%) (Additional file 2: Figure S1).

**Fig. 1:**
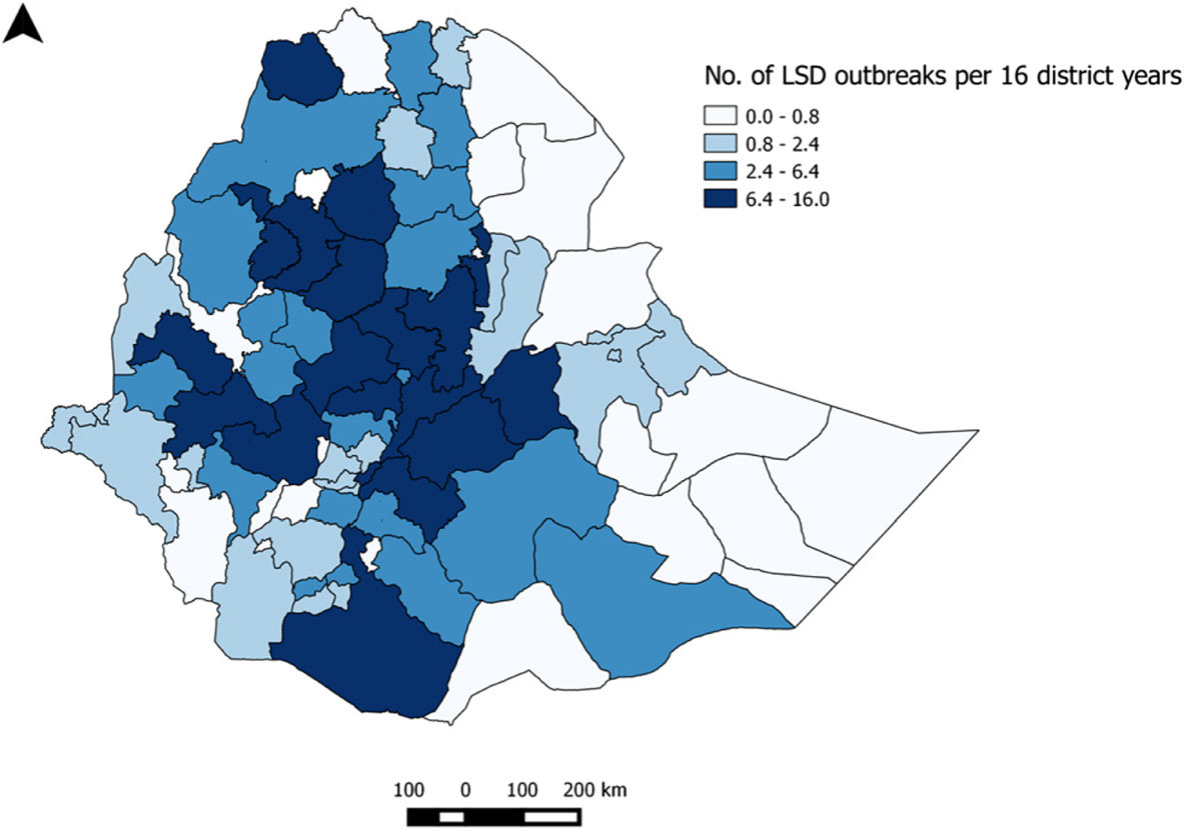
Zonal distribution of LSD outbreaks per 16 district years in Ethiopia over the period 2000–2015

The average incidence of LSD outbreaks at district level was 5.58 over all 16 years or 0.35 per district per year. The lowest incidences were observed in the eastern lowland (Afar and Somali), southern lowland (Liben), south-west (Benchi Maji) and North (North western zone of Tigray) areas whereas the highest number of outbreaks were documented in the north-west, central, West and south-western parts of the country (Fig. 1).

The data shows that LSD affects districts for one or two years and then spreads to other nearby districts/areas with a susceptible cattle population. In this fashion the disease moves from one geographical area to the other and circulates in the country (Additional file 3: Figure S2). The reoccurrence of the disease in the study districts varies from 1 year to 13 years, with average length of 4.54 years and median 4 years. The time between outbreaks was shorter in districts geographically located in the West, south-west and central part of the country.

### LSD outbreak time series description and analysis

**Fig. 2:**
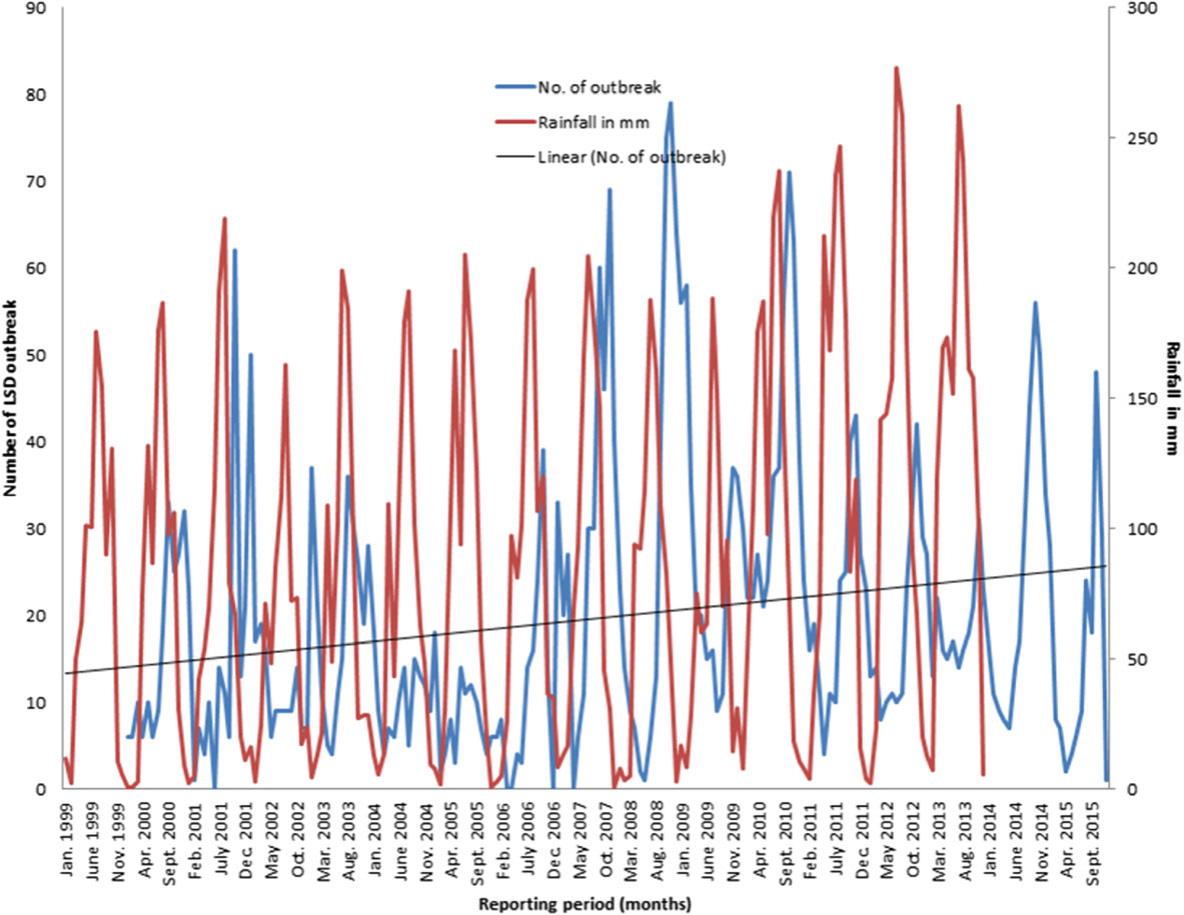
Monthly outbreak and trend of LSD from 2000 to 2015 and monthly rainfall in millimetre from 1999 to 2013 in Ethiopia

**Fig. 3:**
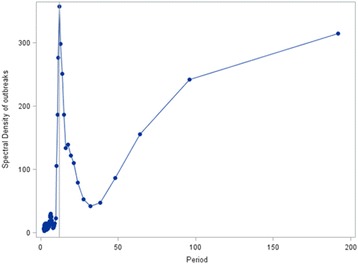
Spectral density estimates of LSD outbreaks by month, the vertical reference line at the 12 month period shows the seasonality of the disease

The monthly distribution of LSD outbreaks is presented in Fig. 2, Additional file 1: Table S1 and Additional file 4: Figure S3. It showed a slight increase in the number of monthly outbreaks which was statistically significant (*P* < 0.05) (Fig. 2). The seasonality in the numbers of outbreaks is apparent, which tend to be higher in the months following the long rainy season compared to other seasons (Fig. 2). The undecomposed and undifferenced original LSD outbreak time series was found seasonal by spectral analysis techniques (Fig. 3).

**Fig. 4:**
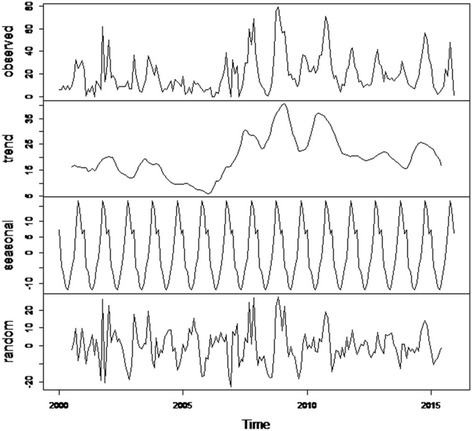
Decomposition of the time series of the number of LSD outbreaks (top panel) into three components: trend, seasonality and random

The trend, seasonal and irregular components of the LSD outbreak time series were estimated by decomposing the time series (Fig. 4). The estimated trend component shows a decrease from about 20 outbreaks in 2002 to about 6 outbreaks in 2006, followed by a substantial increase to about 41 outbreaks in 2009, decrease to about 16 outbreaks at the end of 2013 and finally increase to about 26 outbreaks in 2014. Though the LSD outbreak pattern from the trend component appears to have a cycle with a periodicity of 5–7 years (peaks in 2002, 2009 and 2014) (Fig. 4) it was not established by spectral analysis. Linear regression on the trend component of the decomposed time series shows a statistically significant (*p* < 0.001) increase in monthly LSD outbreak numbers between 2000 and 2015.

**Fig. 5:**
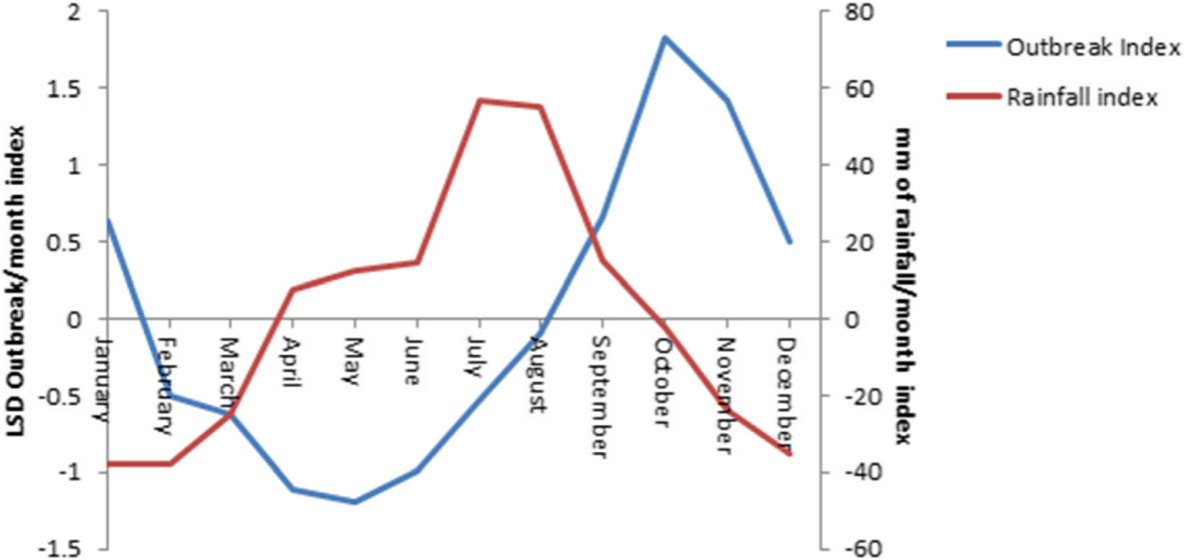
Seasonal indices of monthly LSD outbreak between 2000 and 2015 and mean rainfall between 1999 and 2013 in Ethiopia

The seasonal pattern of LSD outbreaks is clearly indicated in Figs. 3 and 4. Seasonal factors were estimated for each month over the 16 year period as the seasonal component of the decomposed LSD time series. The largest seasonal factor is recorded for October (about 16.8) and the lowest for May (about −12.1), indicating that number of LSD outbreaks peaks in October and has a low (trough) in May (Figs. 4 and 5). In general the number of LSD outbreaks was above average for the months September to January and below average for February to August (Fig. 5).

The rainfall season of Ethiopia is indicated in Figs. 2 and 5. The precipitation is above average from April to September and below average from October to March. The rainfall is high in July and August and low in December to February. The precipitation pattern is the reverse of LSD outbreak pattern (Fig. 5), resulting in a negative correlation coefficient (*r* = −0.05, *p* = 0.49, Spearman rank correlation) at lag 0 and positive correlation coefficients when the series were lagged by 1 to 6 months, the correlation at lag 3 being the highest (*r* = 0.55, *p* < 0.001).

### LSD outbreak times series forecasting

For forecasting with Holt-Winters exponential smoothing, the three parameters: alpha, beta, and gamma which are important for forecasting future values were 0.56, 0.00, and 0.32, respectively. The original LSD outbreak times series and the forecasted values plotted using Holt’s exponential smoothing is shown in Additional file 5: Figure S4. The future times, from January 2016 to December 2018 were also forecasted with Holt-Winters’ exponential methods (Additional file 6: Figure S5). However, the correlogram and Ljung-Box test showed the presence of significant (*P* = 0.002) autocorrelations of the in-sample forecast errors at lags 1–20. This indicates that Holt-Winters exponential smoothing could not provide an adequate forecast.

**Fig. 6:**
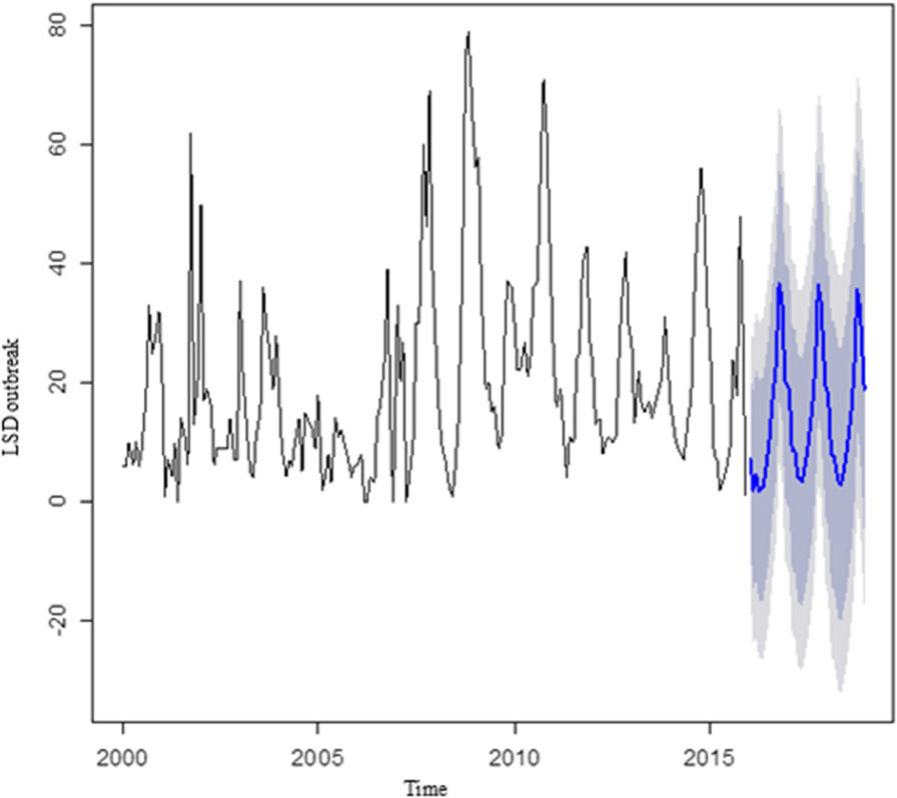
The original LSD outbreak time series (2000–2015) and predicted for the next three years (2016–2018) using ARIMA (1,1,1) x (1,1,1)12 model. The black line represents the original LSD outbreak time series and the blue line the forecasted value. The dark grey shaded area indicate the 80% confidence interval and the light grey 95% confidence interval of the predicted values

The LSD outbreak time series was differenced for trend and seasonality, and the resulting series of first order differences appeared to be stationary in mean and variance. The ACF correlogram of first differenced LSD time series indicates significant autocorrelation at lag 1 (−0.349), 12 (−0.468), and 13 (0.273) (Additional file 7: Figure S6A). This demonstrates the seasonality of the series because the current monthly value is related to the value of 12 months earlier. The partial correlogram also shows that the partial autocorrelations at lags 1 (−0.349), 9 (−0.199), 12 (−0.487) and 15 (0.185) exceed the significance bounds (Additional file 7: Figure S6B). Hence, the ARIMA model (1, 1, 1) x (1, 1, 1)12 was used for making forecasts for the number of LSD outbreaks from January 2016 to December 2018 (Fig. 6). The correlogram for the forecasted value shows that none of the sample autocorrelations for lags 1–20 exceed the significance bounds, and the *p*-value for the Ljung-Box test is 0.107, so it can be concluded that there is little evidence for non-zero autocorrelations in the forecast errors at lags 1–20. Based on the forecast, the highest numbers of LSD outbreaks are expected in October for all predicted years and the lowest in April for 2016 and May for 2017 and 2018 (Additional file 8: Table S2).

## Discussion

In the current study, LSD has been recorded from all regional states and city administrations in Ethiopia. A previous retrospective study that covered a period from January 2007 to December 2011 reported no outbreaks from Dire Dawa city administration and Harari regional state [15]. The present study, however, showed that they are affected by the disease.

Our spatial analysis have shown that distribution of incidence of LSD outbreaks vary among areas (Fig. 1). The highest LSD incidences were in warm moist highland and the lowest in hot dry lowland areas. This indicate that the parts of the country which receive relatively high rain fall for a reasonable period of time is conducive for the replication and survival of blood feeding arthropods and then for the spread of the disease in the geographical areas [7, 15, 30, 31]. The LSD outbreak incidence indicated for the different zones should be treated consciously because under reporting might result in an underestimated incidence.

In this study, it became clear that the occurrence of LSD in an area/districts is sporadic. However, endemicity of the disease is maintained in the country because the outbreaks in different districts/area do not occur at the same time (Additional file 3: Figure S2). The average time to reoccurrence was 4.54 years, in line with the 5 yearly reoccurrence of LSD epidemic in unvaccinated populations [31]. The reoccurrence was variable across the study districts. Some districts reported outbreaks after 1 year of quiescence, whereas others reported an outbreak after a much longer period (up to 13 years), which is in line with Gari et al. [7]. This indicates that the disease is not endemic in a district/an area but it occurs in an outbreak (epidemic) form after some years. The reoccurrence is only possible after the seroprevalence (herd immunity) dropped below the critical value and reproduction ratio (R) is above one. How long it will take depends on the rate at which LSD is introduced into the district/area (spark rate) and how far the R has increased above one [17]. The time between outbreaks was shorter in the West and south-west (where rainfall occurs for extended periods of time) and central (where live animal from different parts of the country cross through to the central market) parts of the country.

LSD outbreaks do also not occur at random in time and we demonstrated the seasonality by spectral analysis (Fig. 3) and estimated a significant autocorrelation between LSD outbreaks at lag 12, 24, 36, etc., indicating the seasonality of the disease. The seasonal pattern of the disease is also clearly indicated in Fig. 4. The seasonal LSD outbreak variation might be related to the variation in temperature and rainfall between seasons leading to varying arthropod densities in the environment. Seasonal variations in vector abundance, including mosquitoes, ticks, and flies, are well known causes of seasonality for vector borne diseases [10]. Identification of temporal patterns can indicate times when particular attention should be paid to control the disease [9].

The trend of LSD outbreaks from January 2000 to December 2015 indicates a slight, but statistically significant increase over the period (Figs. 2 and 4). This might be attributed to the absence of a specific national strategy for LSD control or eradication [18] and the increased tendency of using irrigation for crop cultivation that create favourable environmental conditions for vector borne diseases in the country. The implication of the trend component is that the disease will continue to persist if the environmental circumstances and the poor disease control activities continue.

The positive and significant cross correlations between precipitation and increased LSD outbreaks at lag 3 (*r* = 0.55, *p* < 0.001) suggests that the rainfall in the previous months are an important factor for the occurrence of LSD outbreaks. The time delay for LSD outbreaks to occur might be justified by the time required for the build-up of arthropod population following the rains [32, 33], incubation period (2–4 weeks [34]) of the virus within the cattle host and delay in reporting.

Based on the 2000–2015 reports, the number of LSD outbreaks to occur in each month from 2016 to 2018 was forecasted. The forecast suggests that high number of LSD outbreak will occur from August to January and this is comparable with the available LSD outbreak time series data. The reappearance of the seasonality in the original time series again in the forecasts is an indication of the forecast is reasonable [27]. The wide confidence interval (Fig. 6) indicates the need of frequent updating of the model by incorporating the latest outbreak reports [27]. The confidence interval was even wider when Holt-Winters’ exponential methods were used (Additional file 6: Figure S5). The wider confidence interval is related to a limitation of this method, i.e. it does not take the correlations between successive values of the time series into account. The ARIMA model, taking the correlations in the data into account, therefore, is the preferred model to get a reasonable forecast in this study [26]. The forecasting process can be continued to any point in the future, but will become less reliable for predictions further in time [29]. This means we can only gain advantage from the use of short term forecasts. The forecasted results of this study, therefore, will alert and help policy makers to focus on the unusual situations to decide whether any disease control intervention is required to halt the occurrence of the disease in the future.

Currently, Ethiopia has no a well-designed control strategy for LSD [18]. The animal health authority undertake reactive vaccination campaign using Kenyan sheep pox vaccine when an LSD outbreak is reported somewhere in the country. Vaccination is the only measure taken for LSD control. However, research findings indicate that the vaccine used in Ethiopia is not fully protective [35] which might be the reason for the increase in incidence of LSD outbreaks observed over the current study period. Because there is no regular vaccination program against LSD this might attribute to a drop of herd immunity below the critical point and for the reoccurrence of the disease. We now understood that LSD does not establish endemicity in an area, but it recurs as epidemic in, at average, every 5 years. Therefore, outbreaks might be prevented by bringing the herd immunity above the critical level through vaccination and by prohibiting the entrance of infected animals to the provisionally free area. Vaccination should be undertaken regularly ahead of the onset of the main rainy season with a high coverage. The vaccine currently in use shall be replaced by more competent homologous (Neethling virus) vaccine [36]. It is widely agreed that vaccination is the most manageable and realistic approach to control the disease in endemic and resource poor countries. However, to be more effective, the vaccination should be complemented by other additional measures such as movement control.

## Conclusion

LSD is wide spread and well established in Ethiopia. It occurred in all regional states and city administrations in the time period between 2000 and 2015. LSD does not establish endemicity in a district, but it does in the country as a whole. It recurs in a district as epidemic, on average in 5 years period. The average incidence of LSD outbreaks at district level was 5.58 over all 16 years. The trend of LSD outbreaks increased over time. Outbreaks are seasonal and occurred more often in the months following the long rainy season. The results of the spatiotemporal analysis and the forecasted value may serve as a guide for the routine surveillance of LSD in the country.

## Additional files

Additional file 1: Table S1. Number of LSD outbreaks reported monthly over the period 2000–2015 in Ethiopia. (DOCX 17 kb)

Additional file 2: Figure S1. Distribution of LSD outbreaks (n = 3811) over regional states and city administrations in the period 2000–2015. (DOCX 14 kb)

Additional file 3: Figure S2. Animation of the spread of lumpy skin disease epidemics in Ethiopia, 2000–2015. (DOCX 2749 kb)

Additional file 4: Figure S3. Annual course of LSD outbreaks in Ethiopia, 2000–2015. (DOCX 169 kb)

Additional file 5: Figure S4. The original LSD outbreak time series (black) and the predicted values (red) using Holt-Winters filtering. (DOCX 27 kb)

Additional file 6: Figure S5. LSD outbreak forecasts based on Holt-Winters analysis for January 2016 to December 2018. (DOCX 24 kb)

Additional file 7: Figure S6. ACF (A) and Partial ACF (B) correlogram after first order seasonal and trend differencing of the original LSD outbreak time series. (DOCX 21 kb)

Additional file 8: Table S2. 36 month forecast of the number of LSD outbreaks based on ARIMA (1, 1, 1) x (1, 1, 1)12. (DOCX 17 kb)

## Abbreviations

ACF: Autocorrelation Function
ARIMA: Autoregressive Integrated Moving Average
LSD: Lumpy Skin Disease
OIE: Office International des Epizooties (world organization for animal health)
R: Reproduction ratio
SAS: Statistical Analysis Software
SNNP: Southern Nations Nationalities and Peoples Region
SPMAP: South Platte Mapping and Analysis program
SWAT: Soil, Water, and Air Team
TTR: Technical Trading Rules
