# Supplementary material for: Temporal and spatial distribution of lumpy skin disease outbreaks in Ethiopia in the period 2000 to 2015
Source: BMC Vet Res. 2017 Nov 6;13:310. doi: 10.1186/s12917-017-1247-5 (PMC5674741; doi:10.1186/s12917-017-1247-5)
Supplement: Supplementary file 1 — Number of LSD outbreaks reported monthly over the period 2000–2015 in Ethiopia. (DOCX 17 kb) [file 12917_2017_1247_MOESM1_ESM.docx]

Table S1. Number of LSD outbreaks reported monthly over the period 2000-2015 in Ethiopia.

| Year | Number of LSD outbreaks | | | | | | | | | | | | |
| --- | --- | --- | --- | --- | --- | --- | --- | --- | --- | --- | --- | --- | --- |
|  | Jan. | Feb. | Mar. | Apr. | May | Jun. | Jul. | Aug. | Sep. | Oct. | Nov. | Dec. | Year total |
| 2000 | 6 | 6 | 10 | 6 | 10 | 6 | 9 | 18 | 33 | 25 | 27 | 32 | 188 |
| 2001 | 23 | 1 | 7 | 4 | 10 | 0 | 14 | 11 | 6 | 62 | 13 | 21 | 172 |
| 2002 | 50 | 17 | 19 | 16 | 6 | 9 | 9 | 9 | 9 | 14 | 7 | 7 | 172 |
| 2003 | 37 | 21 | 11 | 5 | 4 | 11 | 15 | 36 | 30 | 26 | 19 | 28 | 243 |
| 2004 | 18 | 9 | 4 | 7 | 6 | 10 | 14 | 5 | 15 | 13 | 12 | 9 | 122 |
| 2005 | 18 | 2 | 4 | 8 | 3 | 14 | 11 | 12 | 10 | 7 | 4 | 6 | 99 |
| 2006 | 6 | 8 | 0 | 0 | 4 | 3 | 14 | 16 | 24 | 39 | 14 | 0 | 128 |
| 2007 | 33 | 20 | 27 | 0 | 6 | 11 | 30 | 30 | 60 | 46 | 69 | 40 | 372 |
| 2008 | 23 | 14 | 9 | 7 | 2 | 1 | 6 | 13 | 40 | 75 | 79 | 64 | 333 |
| 2009 | 56 | 58 | 35 | 19 | 20 | 15 | 16 | 9 | 11 | 27 | 37 | 36 | 339 |
| 2010 | 30 | 22 | 22 | 27 | 21 | 24 | 36 | 37 | 56 | 71 | 63 | 38 | 447 |
| 2011 | 24 | 16 | 19 | 11 | 4 | 11 | 10 | 24 | 25 | 40 | 43 | 27 | 254 |
| 2012 | 23 | 13 | 14 | 8 | 10 | 11 | 10 | 11 | 23 | 33 | 42 | 29 | 227 |
| 2013 | 27 | 13 | 22 | 16 | 15 | 17 | 14 | 16 | 18 | 21 | 31 | 23 | 233 |
| 2014 | 16 | 11 | 9 | 8 | 7 | 14 | 17 | 32 | 44 | 56 | 50 | 34 | 298 |
| 2015 | 28 | 8 | 7 | 2 | 4 | 6 | 9 | 24 | 18 | 48 | 29 | 1 | 184 |
| Average | 26.1 | 14.9 | 13.7 | 9 | 8.3 | 10.2 | 14.6 | 18.9 | 26.4 | 37.7 | 33.7 | 24.7 | 238.2 |
