# Supplementary material for: Temporal and spatial distribution of lumpy skin disease outbreaks in Ethiopia in the period 2000 to 2015
Source: BMC Vet Res. 2017 Nov 6;13:310. doi: 10.1186/s12917-017-1247-5 (PMC5674741; doi:10.1186/s12917-017-1247-5)
Supplement: Supplementary file 2 — Distribution of LSD outbreaks (n = 3811) over regional states and city administrations in the period 2000–2015. (DOCX 14 kb) [file 12917_2017_1247_MOESM2_ESM.docx]

Figure S1. Distribution of LSD outbreaks (n = 3811) over regional states and city administrations in the period 2000-2015.
