# Supplementary figures and images for: Temporal and spatial distribution of lumpy skin disease outbreaks in Ethiopia in the period 2000 to 2015
Source: BMC Vet Res. 2017 Nov 6;13:310. doi: 10.1186/s12917-017-1247-5 (PMC5674741; doi:10.1186/s12917-017-1247-5)

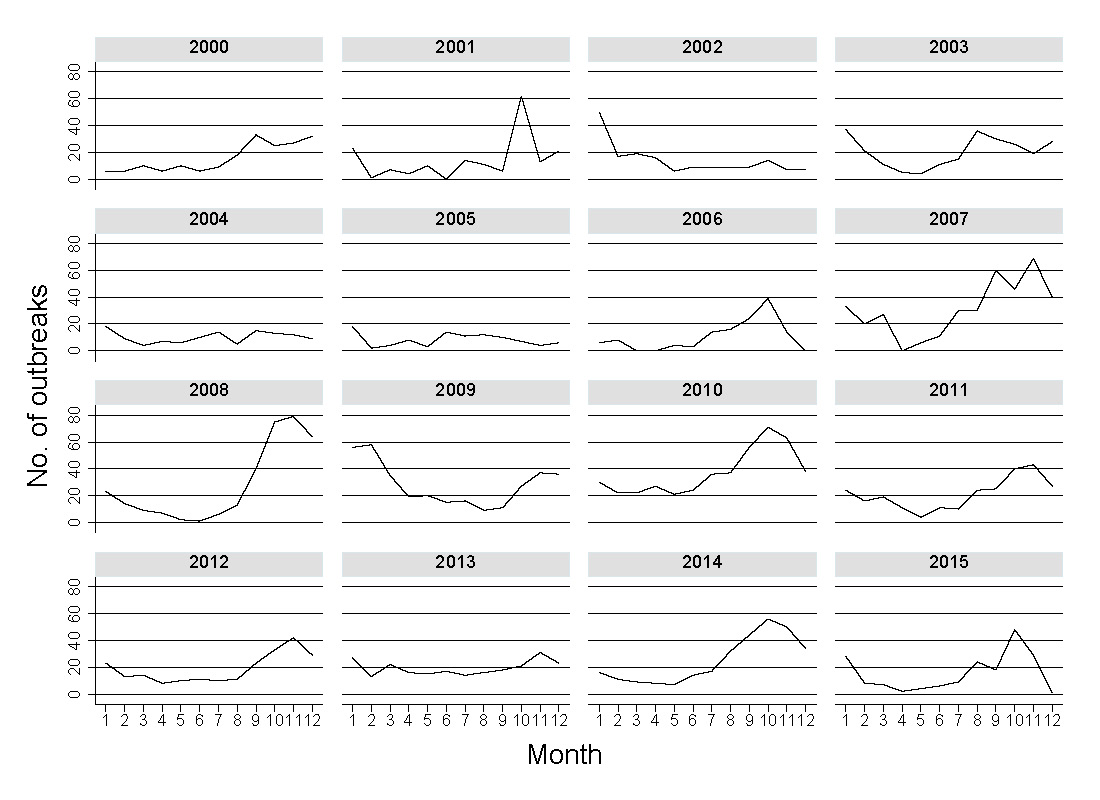


Figure S3. Annual course of LSD outbreaks in Ethiopia, 2000-2015.

Supplement: Supplementary file 4 — Annual course of LSD outbreaks in Ethiopia, 2000–2015. (DOCX 169 kb) [file 12917_2017_1247_MOESM4_ESM.docx]
