# Supplementary material for: Temporal and spatial distribution of lumpy skin disease outbreaks in Ethiopia in the period 2000 to 2015
Source: BMC Vet Res. 2017 Nov 6;13:310. doi: 10.1186/s12917-017-1247-5 (PMC5674741; doi:10.1186/s12917-017-1247-5)
Supplement: Supplementary file 5 — The original LSD outbreak time series (black) and the predicted values (red) using Holt-Winters filtering. (DOCX 27 kb) [file 12917_2017_1247_MOESM5_ESM.docx]

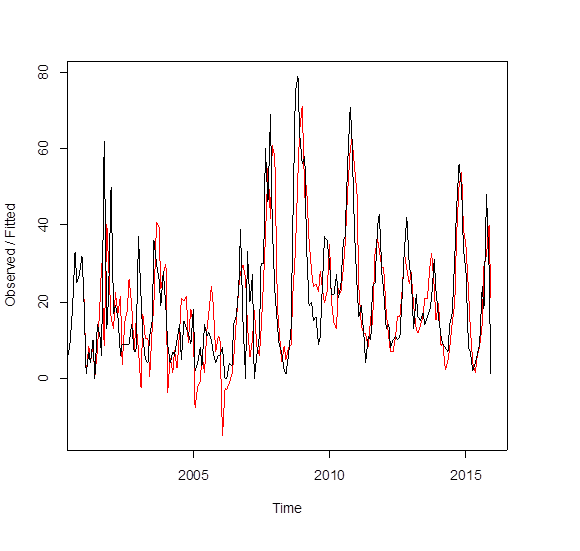


Figure S4. The original LSD outbreak time series (black) and the predicted values (red) using Holt-Winters filtering.
