# Supplementary material for: Temporal and spatial distribution of lumpy skin disease outbreaks in Ethiopia in the period 2000 to 2015
Source: BMC Vet Res. 2017 Nov 6;13:310. doi: 10.1186/s12917-017-1247-5 (PMC5674741; doi:10.1186/s12917-017-1247-5)
Supplement: Supplementary file 6 — LSD outbreak forecasts based on Holt-Winters analysis for January 2016 to December 2018. (DOCX 24 kb) [file 12917_2017_1247_MOESM6_ESM.docx]

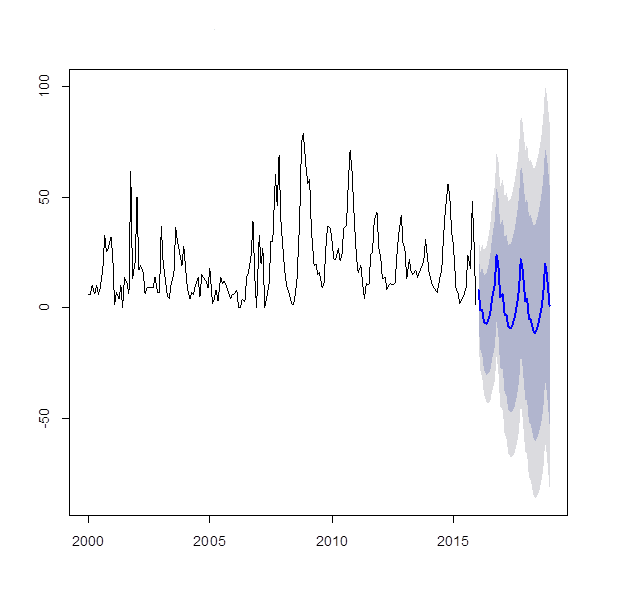


Figure S5. LSD outbreak forecasts based on Holt-Winters analysis for January 2016 to December 2018.

The forecasts are shown as a blue line, and the dark grey and light grey shaded areas show 80% and 95% prediction intervals, respectively.
