# Supplementary material for: Temporal and spatial distribution of lumpy skin disease outbreaks in Ethiopia in the period 2000 to 2015
Source: BMC Vet Res. 2017 Nov 6;13:310. doi: 10.1186/s12917-017-1247-5 (PMC5674741; doi:10.1186/s12917-017-1247-5)
Supplement: Supplementary file 7 — ACF (A) and Partial ACF (B) correlogram after first order seasonal and trend differencing of the original LSD outbreak time series. (DOCX 21 kb) [file 12917_2017_1247_MOESM7_ESM.docx]

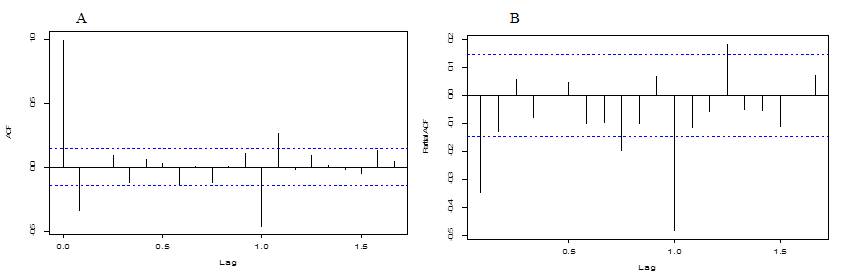


Figure S6. ACF (A) and Partial ACF (B) correlogram after first order seasonal and trend differencing of the original LSD outbreak time series
