# Supplementary material for: Temporal and spatial distribution of lumpy skin disease outbreaks in Ethiopia in the period 2000 to 2015
Source: BMC Vet Res. 2017 Nov 6;13:310. doi: 10.1186/s12917-017-1247-5 (PMC5674741; doi:10.1186/s12917-017-1247-5)
Supplement: Supplementary file 8 — 36 month forecast of the number of LSD outbreaks based on ARIMA (1, 1, 1) x (1, 1, 1)12. (DOCX 17 kb) [file 12917_2017_1247_MOESM8_ESM.docx]

Table S2. 36 month forecast of the number of LSD outbreaks based on ARIMA (1, 1, 1) x (1, 1, 1)12.

| **Month** | **Point Forecast** | **Lo 80** | **Hi 80** | **Lo 95** | **Hi 95** |
| --- | --- | --- | --- | --- | --- |
| Jan 2016 | 7.18 | -6.66 | 21.02 | -13.99 | 28.35 |
| Feb 2016 | 1.95 | -14.69 | 18.60 | -23.50 | 27.41 |
| Mar 2016 | 4.63 | -13.20 | 22.47 | -22.64 | 31.91 |
| Apr 2016 | 1.85 | -16.58 | 20.29 | -26.34 | 30.05 |
| May 2016 | 2.32 | -16.46 | 21.11 | -26.40 | 31.05 |
| Jun 2016 | 5.76 | -13.25 | 24.77 | -23.36 | 34.83 |
| Jul 2016 | 9.43 | -9.75 | 28.61 | -19.90 | 38.76 |
| Aug 2016 | 16.01 | -3.30 | 35.33 | -13.53 | 45.55 |
| Sep 2016 | 23.21 | 3.78 | 42.65 | -6.50 | 52.93 |
| Oct 2016 | 36.57 | 17.03 | 56.10 | 6.68 | 66.45 |
| Nov 2016 | 34.15 | 14.51 | 53.78 | 4.11 | 64.18 |
| Dec 2016 | 20.39 | 0.66 | 40.13 | -9.79 | 50.57 |
| Jan 2017 | 18.87 | -1.25 | 38.99 | -11.90 | 49.64 |
| Feb 2017 | 8.75 | -11.63 | 29.13 | -22.41 | 39.92 |
| Mar 2017 | 8.54 | -12.03 | 29.11 | -22.92 | 40.00 |
| Apr 2017 | 3.98 | -16.75 | 24.71 | -27.73 | 35.68 |
| May 2017 | 3.42 | -17.45 | 24.29 | -28.50 | 35.34 |
| Jun 2017 | 6.17 | -14.83 | 27.17 | -25.94 | 38.28 |
| Jul 2017 | 9.42 | -11.70 | 30.54 | -22.87 | 41.72 |
| Aug 2017 | 15.94 | -5.30 | 37.17 | -16.54 | 48.41 |
| Sep 2017 | 22.71 | 1.36 | 44.05 | -9.94 | 55.35 |
| Oct 2017 | 36.32 | 14.86 | 57.77 | 3.50 | 69.13 |
| Nov 2017 | 33.49 | 11.92 | 55.06 | 0.51 | 66.47 |
| Dec 2017 | 19.40 | -2.28 | 41.08 | -13.75 | 52.55 |
| Jan 2018 | 18.02 | -4.10 | 40.14 | -15.81 | 51.85 |
| Feb 2018 | 7.99 | -14.43 | 30.41 | -26. 30 | 42.28 |
| Mar 2018 | 7.83 | -14.81 | 30.48 | -26.80 | 42.47 |
| Apr 2018 | 3.30 | -19.53 | 26.13 | -31.62 | 38.22 |
| May 2018 | 2.76 | -20.23 | 25.76 | -32.41 | 37.93 |
| Jun 2018 | 5.53 | -17.62 | 28.67 | -29.87 | 40.92 |
| Jul 2018 | 8.78 | -14.50 | 32.07 | -26.83 | 44.40 |
| Aug 2018 | 15.30 | -8.12 | 38.72 | -20.52 | 51.12 |
| Sep 2018 | 22.08 | -1.48 | 45.64 | -13.95 | 58.11 |
| Oct 2018 | 35.68 | 12.00 | 59.37 | -0.54 | 71.91 |
| Nov 2018 | 32.86 | 9.05 | 56.68 | -3.56 | 69.29 |
| Dec 2018 | 18.78 | -5.16 | 42.73 | -17.84 | 55.40 |
