# Supplementary material for: Temporal and spatial distribution of lumpy skin disease outbreaks in Ethiopia in the period 2000 to 2015
Source: BMC Vet Res. 2017 Nov 6;13:310. doi: 10.1186/s12917-017-1247-5 (PMC5674741; doi:10.1186/s12917-017-1247-5)
Supplement: Supplementary file 8 [file 12917_2017_1247_MOESM3_ESM.docx]

Figure S2. Animation of the spread of lumpy skin disease epidemics in Ethiopia, 2000-2015

Wassie Molla Abebe
Faculty of Veterinary Medicine / Quantitative Veterinary Epidemiology group
University of Gondar /Wageningen University & Research
Ethiopia / Wageningen, The Netherlands
